# PReS-FINAL-2087: Diagnosis of arthritis: history or laboratory?

**DOI:** 10.1186/1546-0096-11-S2-P99

**Published:** 2013-12-05

**Authors:** O Yurtsever, K Gulleroglu, US Bayrakci, A Kantar, E Baskin

**Affiliations:** 1 PedNephrology and Rheumatology, Baskent University, Ankara, Turkey

## Introduction

Arthritis is a common rheumatologic problem of childhood. Physical examination and laboratory findings are important to clarify the etiology.

## Objectives

We evaluated retrospectively patients with arthritis for determine the most beneficial findings in the diagnosis.

## Methods

151 patients (82 girls and 69 boys) were included to the study. Demographic characteristics of the patients and laboratory parameters were recorded. All patients underwent detailed physical assessment. Ophthalmological examination and imaging studies were performed in selected patients.

## Results

Median age was 10 years (range 1-17). Diagnoses of the patients were Juvenile Idiopathic Arthritis (JIA) (35.7), reactive arthritis (15%), Familial Mediterranean Fever (FMF) (12.7%), connective tissue disorder/vasculitis (11.5%) and septic arthritis (3.8%). The remaining of patients (15.9%) was classified as arthritis of unknown etiology. Of all patients 51% had monoarticular, 38% had oligoarticular and 11% had polyarticular involvement.

41% of JIA patients had monoarthritis, 42% of them had oligoarthritis. Polyarthritis was detected in 16% of JIA cases. 75% of FMF patients had monoarticular arthritis. Sacroiliac tenderness was detected in 33 patients (25.2%). Seventeen of these patients were diagnosed as JIA. Uveitis was defined in 9% of patients. Diagnoses of patients with uveitis were JIA (71%) and connective tissue disorder/vasculitis (29%). Ophthalmological examination was normal in other disease groups (p < 0.05). Patients with uveitis had mostly monoarticular arthritis (50%).

HLA B27 was positive in 6 patients (4%). Sacroileitis was determined in 66% of those patients. ANA was positive in 25% of patients and only 43.9% of patients with JIA had positive test for ANA. There was no significant difference in levels of acute phase reactants in different disease groups.

## Conclusion

Patients with monoarticular involvement, uveitis and sacroileitis are tended to be diagnosed as JIA, and this finding correlates with previous studies. Laboratory findings are not always beneficial in the diagnosis of the rheumatologic diseases. Physical examination and a detailed history are more important for diagnosis and these will be helpful in the future as well.

## Disclosure of interest

None declared.

